# Critical Overview of the Risk Scoring Systems to Predict Non-Responsiveness to Intravenous Immunoglobulin in Kawasaki Syndrome

**DOI:** 10.3390/ijms17030278

**Published:** 2016-02-24

**Authors:** Donato Rigante, Laura Andreozzi, Michele Fastiggi, Benedetta Bracci, Marco Francesco Natale, Susanna Esposito

**Affiliations:** 1 Institute of Pediatrics, Università Cattolica Sacro Cuore, Fondazione Policlinico Universitario A. Gemelli, Rome 00168, Italy; laurandreozzi@gmail.com (L.A.); micstiggi@gmail.com (M.F.); benedetta.bracci@gmail.com (B.B.); mark000@hotmail.it (M.F.N.); 2 Pediatric Highly Intensive Care Unit, Department of Pathophysiology and Transplantation, Università degli Studi di Milano, Fondazione IRCCS Ca’ Granda Ospedale Maggiore Policlinico, Milan 20122, Italy; susanna.esposito@unimi.it

**Keywords:** Kawasaki syndrome, intravenous immunoglobulin, coronary artery abnormalities

## Abstract

Kawasaki syndrome (KS) is the most relevant cause of heart disease in children living in developed countries. Intravenous immunoglobulin (IVIG) has a preventive function in the formation of coronary artery abnormalities and a poor strictly-curative action in established coronary damage. More than two decades ago, the Harada score was set to assess which children with KS should be subject to administration of IVIG, evaluating retrospectively a large cohort of patients with regard to age, sex and laboratory data. Nowadays, high dose IVIG is administered to all children with a confirmed diagnosis of KS, but a tool for predicting non-responsiveness to the initial infusion of IVIG has not been found. The prediction of IVIG resistance is a crucial issue, as recognising these high-risk patients should consent the administration of an intensified initial treatment in combination with IVIG in order to prevent coronary injuries. Few reports have focused on factors, referring to both clinical parameters and laboratory data at the onset of KS, in order to predict which patients might be IVIG non-responsive. We have analysed three different risk scores which were formulated to predict IVIG resistance in Japanese children with typical KS, but their application in non-Japanese patients or in those with incomplete and atypical patterns of the disease has been studied in a fragmentary way. Overall, our analysis showed that early and definite ascertainment of likely IVIG non-responders who require additional therapies reducing the development of coronary artery involvement in children with KS is still a challenge.

## 1. Introduction

Almost half a century has passed since Kawasaki syndrome (KS) was first reported by Tomisaku Kawasaki as an enigmatic disease affecting children, and, today, KS still remains uncannily dangerous due to the intrinsic risk of damaging the vascular system, mostly the coronary arteries, in 25% of untreated patients. It is no coincidence that KS stands as the most relevant cause of heart disease in children of developed countries, not only in Japan where the disease is largely observed and recognized [1,2]. Many shortcomings still exist in studies aimed at defining the etiology of KS, which makes it an arduous task to improve the recommendations given in KS treatment [3,4]. Though a host of clinicians around the world have invested huge effort to unveil the mysteries of KS, studies related to the delineation of its outcomes remain lacking. The most dangerous manifestations of KS are not part of the diagnostic criteria, and include myocarditis, congestive heart failure and coronary artery aneurysms. Prompt treatment with intravenous immunoglobulin (IVIG) has been shown to resolve all manifestations of KS and to significantly decrease the risk of development of coronary artery abnormalities (CAA) [5]. Actual therapeutic management, which is centred on the administration of IVIG within 10 days of the onset of fever, has been categorised according to severity of CAA and the type of subsequent cardiovascular risk [6]. Indeed, if a patient responds to IVIG, there is a better chance to decrease the risk of KS-induced cardiovascular complications from 20% to as low as 5% [7]. Many recent data suggest that treatment and prognostic issues are dissociated with the etiology of KS, though the extent of acute phase response and a younger age at onset are probably related to patients’ responsiveness to IVIG [8]. The main aim of this review was to analyse the three more recent risk scores which were formulated to predict IVIG resistance in Japanese populations of children with typical KS, and to evaluate their pertinence in non-Japanese patients or in those with incomplete and atypical patterns of the disease.

## 2. The Harada Score in Kawasaki Syndrome

Methods to predict which children are at greatest risk of developing CAA have been extensively sought to determine the general prognosis of KS. The first scoring model was formulated in 1991 by Harada, but it was not intentionally created to assess a general risk of cardiovascular involvement: it merely tried to identify KS children at higher risk for potential complications, as different issues related to cost and availability of IVIG made it problematic at that time to treat all children with KS in Japan. The “Harada score” was developed by retrospectively correlating routine laboratory and clinical findings in the early stages of KS, *i.e.*, within nine days of onset, with the eventual development of CAA. The study was set in two parts: the first part aimed to evaluate doses and types of IVIG, the second aimed to assess the elective indications of IVIG for children with KS. In particular, Harada analysed data from 865 naïve patients who had not been treated with IVIG, corticosteroids or nonsteroidal anti-inflammatory drugs, and the scoring criteria included seven items: white blood cell and platelet counts, C-reactive protein (CRP), hematocrit, albumin, age at diagnosis, and male sex. Children with KS who satisfied at least four of these seven criteria were thought to be candidates for IVIG treatment (List 1) [9].

List 1. The Harada score (1991) was extrapolated by a multicentre study carried out to evaluate the effectiveness of different doses and kinds of intravenous globulin (IVIG) in 865 naïve patients with Kawasaki syndrome, analysed retrospectively with regard to laboratory data, age, and sex: the score was an attempt to establish a set of criteria for starting treatment with IVIG.

The Presence of at Least 4 of these 7 Criteria prompted the Administration of Intravenous Immunoglobulin to the Child with Kawasaki Syndrome:

- White blood cell count > 12 × 10^3^/mm^3^
- Platelet count < 35 × 10^4^/mm^3^
- C-reactive protein > 3+
- Hematocrit < 35%
- Albumin < 3.5 g/dL
- Age < 12 months
- Sex Male

Although this score should not be used to triage children who do not need IVIG, its influence was dispelled over time because, nowadays, IVIG is necessarily given to “all” KS patients. In 2014 Tewelde *et al.* [10] performed a retrospective chart review of 105 patients admitted to the Cleveland Children’s Clinic from 2001 to 2011 whose final diagnosis was KS, and suggested that the Harada score was effective also in the USA population (having a median age at time of diagnosis of 2.8 years), with the aim of selecting high-risk children who might benefit from further evaluation and additional therapies beyond standard IVIG. They also concluded that the score had adequate sensitivity (90%) in the selection of children with higher risk of developing CAA, though low specificity (51%).

## 3. The Risk Scores for the Evaluation of Non-Responsiveness to Intravenous Immunoglobulin in Kawasaki Syndrome

Different researchers have focused on better recognising early predictors of IVIG resistance with the hope of developing risk scoring algorithms and estimating the probability of a successful response to IVIG. In 2009, Son *et al.* [11] indicated that a percentage of KS patients varying from 13% to 21% were resistant to IVIG, displaying persistent or recrudescent fever after first IVIG administration. Few scoring models to predict the risk of CAA have been formulated. The first of these, reported by Asai in 1983, tried to determine an indication for cardiac catheterization during a period in which echocardiography was not routinely used in Japan in the management of KS [12].

Another scoring model was created by Nakano *et al.* [13] in 1986, starting from clinical and early laboratory findings from 78 children who were hospitalized since the 4th day of fever to the 7th: they found that age at onset, CRP, and baseline platelet count were useful items in the differentiation of patients at higher risk of CAA.

In 1998, Beiser *et al.* [14] constructed a sequential risk classification tool from a cohort of 212 children with KS treated with IVIG during the first 10 days of disease to predict which patients might develop CAA, and evaluate baseline hemoglobin level, neutrophil count, platelet count, and body temperature on the day in which IVIG was infused: the authors distinguished low-risk and high-risk children, among whom extensive cardiac monitoring and even additional therapies could be appropriate.

In 2000, after a multivariate analysis of 82 KS patients, Fukunishi *et al.* [15] noted that those with CRP above 100 mg/L, lactate dehydrogenase above 590 IU/L and/or hemoglobin below 10 g/dL before initial treatment with high-dose IVIG were probably IVIG non-responders; they also noted that baseline total bilirubin was significantly higher in IVIG non-responsive patients by univariate analysis.

The prediction of IVIG resistance has been also evaluated by three more recent risk scores, formulated in the last decade and based on children with typical KS: these three scoring systems have been considered and schematized in Table 1 for this review.

### 3.1. The Egami Score

In 2006, Egami *et al.* [16] defined a prediction score of non-responsiveness to IVIG evaluating through logistic regression analysis the clinical, laboratory and demographic data of 320 Japanese patients, hospitalized in the period 1998–2004, who received IVIG (2 g/kg as a single infusion) by the 9th day of disease, without cardiovascular complications at initial treatment: the number of patients with resistance to IVIG was 41 (13%), while the other 279 patients were full responders. In this model (in which the authors assigned 1 point for age, duration of illness in days, platelet count, CRP and 2 points for serum alanine aminotransferase, and in which a cut-off point ≥3 pinpointed patients with KS at high risk of being resistant to IVIG), the clinician should recognize the IVIG non-responders with 78% sensitivity and 76% specificity, and predict the potential development of CAA with 61% sensitivity and 81% specificity.

However, in the same year, Tremoulet *et al.* [19] (at the University of California, San Diego) evaluated the Egami score in 362 children diagnosed with KS and found that more than 60% of IVIG-resistant patients had been missed.

### 3.2. The Kobayashi Score

Also in 2006, Kobayashi *et al.* [17] created a new model to predict non-responsiveness to IVIG in children with KS, reviewing retrospectively the clinical records of 546 KS patients hospitalized in the period 2000–2004 in 13 Japanese hospitals; a further group of 204 patients hospitalized in the period 2004–2006 was also studied prospectively to confirm accuracy of the previous results. IVIG was given as 1 g/kg per day for 2 days in combination with aspirin (30 mg/kg, then reduced to an anti-platelet dosage after that CRP was normalized) and dipyridamole (2 mg/kg per day). Patients were considered to be non-responders if they were persistently febrile or if they returned with KS symptoms after defervescence. Univariate analysis found 10 laboratory variables (percentage of neutrophils, platelet count, total bilirubin, aspartate and alanine aminotransferases, sodium and chloride, proteins and albumin, and CRP) as likely predictors of IVIG non-responsiveness, in combination with three demographic variables (male sex, age expressed in months, and the number of days of illness until when treatment was started), which were all included in the stepwise forward logistic regression analysis. A risk score was finally developed by the integration of laboratory variables (evaluated before treatment) and demographic data: this model was used to define two risk levels for patients with KS, indicating low- and high-risk of non-responsiveness to IVIG. In particular, they found that a high level of aspartate aminotransferase, CRP, percentage of neutrophils, and also low age in months, low serum sodium and low platelet count, combined with early infusion of IVIG, were all independent risk factors for IVIG non-responsiveness. Sensitivity and specificity in revealing IVIG non-responders were 86% and 67%, respectively, in the logistic model. In the high-risk group (with score >4), the occurrence of IVIG non-responsiveness was 43%, but it was only 5% in the low-risk group (with score <3). The occurrence of CAA was 16% and 1%, respectively, in the high- and low-risk groups. Because KS patients displaying higher scores were at higher risk for the development of CAA, a careful follow-up should be warranted in such patients and a more aggressive initial anti-inflammatory treatment or alternative treatments considered.

### 3.3. The Sano Score

In 2007, Sano *et al.* [18] analysed retrospectively clinical, laboratory, and echocardiographic data of 112 patients with KS aged 1 month to 7 years, hospitalized between 1999 and 2000, to evaluate three potential predictors of non-responsiveness to IVIG: CRP, total bilirubin, and aspartate aminotransferase. This retrospective study was intended to elucidate the correlation between the response to IVIG given within 2 days (1 g/kg/day for two days) and clinical parameters at the onset of KS; the final purpose of the study was to ascertain which children would be non-responsive to the first course of IVIG, for whom alternative intensive primary treatment should be considered. In particular, KS patients displaying the increase of at least two of three predictors at the onset of disease, before IVIG administration, were considered likely non-responders to IVIG; the sensitivity and the specificity of the score were 77% and 86%, respectively. The total incidence of CAA was extremely high (71%) in IVIG non-responsive patients compared to IVIG responders (5%), confirming the potential prediction of CAA at the onset of the disease (earlier than IVIG infusion) by the use of simple biochemical laboratory data. Sano *et al.*’s [18] analysis led to similar results to those obtained in the study by Fukunishi *et al.* [15], who also evaluated the possibility of non-responsiveness to IVIG at disease onset in a cohort of 82 KS patients assessed consecutively in a single Japanese hospital in 2000, choosing body temperature on the 6th day after IVIG administration as an endpoint, instead of CAA: 13 out of 82 patients remained febrile on day 6 after IVIG, and all were characterised by significantly higher values of CRP (>100 mg/L), total bilirubin, lactate dehydrogenase, γ-glutamyl-transpeptidase, and lower values of haemoglobin (<11 g/dL) before treatment.

## 4. A Critical Assessment of the Risk Scoring Systems for Kawasaki Syndrome

All these above-mentioned scores have considered multiple items, which have been listed synthetically in Table 2. High CRP level was globally considered a risk factor for non-responsiveness to IVIG and the subsequent risk of CAA in all systems, probably as a result of stronger systemic inflammation driving endothelial abnormalities and final development of cardiovascular complications. In addition, most scores have considered thrombocytopenia and patient’s age less than 6–12 months as individual risk factors for the occurrence of CAA.

These risk scores, characterised by different sensitivity and specificity, should be judged and scrutinized in light of the different clinical studies in which they were formulated (see Table 3).

First of all, we need to recognise that scores were based on data deriving from Japanese children, and we are all aware that variations in KS incidence or severity of presentation in different populations probably reflect genetic and environmental differences, which makes it problematic to indiscriminately apply these scores to non-Japanese populations. Another relevant issue is the sample size used in each study: Kobayashi and Egami included 750 and 321 patients, respectively [16,17], while Sano considered only 112 patients, with obvious limitations in the study itself [18]. Furthermore, diagnosis of KS was based on different clinical criteria, and only Sano *et al.* [20] assessed CAA adjusting the dimensions of coronary arteries to the patient’s body surface area (by linear regression analysis after comparison with healthy controls). In addition, each study used a different regimen of IVIG dosage. A single IVIG dose of 2 g/kg (the actual standard therapy for KS in Europe, USA, United Kingdom, Australia, and many regions of Asia [21]) was used by Egami *et al.* [17] in combination with daily aspirin (30 mg/kg/day) [16]. Kobayashi *et al.* [17] used 1 g/kg/day of IVIG for two days in combination with aspirin and dipyridamole. Conversely, Sano *et al.* [18] used two separate infusions of IVIG on two consecutive days (at a daily dose of 1 g/kg/day) in the first two days since disease onset in combination with aspirin: in this case, timing of IVIG administration could be an important limitation of the study, as a very early treatment of KS within four days represents an additional risk factor for IVIG non-responsiveness [22]. Indeed, also Egami and Kobayashi demonstrated that untimely administration of IVIG in the first four days of illness might be a further factor contributing to non-responsiveness [16,17].

The last difference among the three studies was the definition of IVIG non-responders: it is known that up to 20% of KS patients might present a persistent or recrudescent fever after IVIG, remaining at high risk of developing CAA [23,24,25]. Egami defined “responder” as a patient in whom fever disappeared and CRP dropped by more than 50% within 48 h after IVIG [16]. Kobayashi defined “non-responder” as a patient who had persistent fever (≥37.5 °C) or a recrudescent fever after an apparent initial defervescence [17]. Finally, Sano defined non-responsiveness to IVIG as persistent fever (≥37.5 °C over 24 h) after completing IVIG infusion [18]. The variable performance of these three scores in the prediction of IVIG resistance in children with KS might be also attributed to all these differences collectively.

In 2011, Sleeper *et al.* [26] performed a randomised trial with pulsed corticosteroids for treatment of KS to assess the properties of the three risk scores primed in Japan, examining whether corticosteroid therapy reduces the risk of CAA in a North American population of KS patients classified as IVIG resistant; the authors found a low sensitivity (42%, 33%, and 40% for the Egami, Kobayashi, and Sano scores, respectively), but a moderate-to-high specificity (85%, 87%, and 85%, respectively). These results suggested that the use of Japanese-based scores in North American children of mixed ethnicity might leave out patients with higher risk who could be ideal candidates for additional therapies to interrupt the disease process.

Until 2015, the benefit of different risk scores to predict IVIG resistance had not been verified in children with an incomplete pattern of KS, which is more prevalent in both infants and older children, for whom the duration of fever is probably longer than in typical KS. In 2015, however, Kanamitzu *et al.* [27] evaluated the performance of Egami, Kobayashi and Sano scores in 51 patients with incomplete KS, reviewing retrospectively the clinical records of those who received IVIG in the period 2005–2012. This study showed that children with incomplete KS were not significantly different between IVIG-resistant and responders according to the Egami, Kobayashi and Sano scores. Conversely, a significant difference was noted for children with a typical KS: in particular, Egami and Kobayashi’s risk scores worked better in the cases of typical KS, while Sano’s score worked better in incomplete KS cases.

One possible reason is that this score did not consider patients’ ages and the overall number of febrile days as risk factors. Another potential reason is that the Sano score includes total bilirubin, suggesting a severe diffuse systemic inflammation just in patients with incomplete KS [27].

## 5. Future Directions in the Evaluation of Children with Kawasaki Syndrome

The relationship between newly recognized laboratory markers and failure to respond to the conventional treatment with IVIG in KS is puzzling. For instance, Huang *et al.* [28] found that serum haptoglobin/apolipoprotein A-I ratio was significantly higher in a cohort of 64 KS patients, and suggested that this ratio could be a supplemental diagnostic marker for differentiating KS from other febrile disorders of childhood. In addition, Demir *et al.* [29] demonstrated that neutrophil-to-lymphocyte ratio (NLR) values, which can be calculated by dividing the neutrophil count by the lymphocyte count, were significantly higher in 75 patients with KS complicated by CAA than in the ones without. In particular, Ha *et al.* [30] evaluated the NLR in 587 patients with KS before IVIG, two days after IVIG (regardless of defervescence), and three to four weeks after defervescence: children who were resistant to IVIG had higher NLRs, and multivariate analysis showed that the NLR calculated two days after IVIG predicted both development of CAA and resistance to IVIG.

Sato *et al.* [31] determined retrospectively the predictors of IVIG resistance in a cohort of 129 children diagnosed with KS in a single-centre: the IVIG-resistant group (consisting of 21 patients) had significantly increased serum levels of interleukin (IL)-6 (207.7 ± 127.1 *versus* 102.7 ± 97.4 pg/mL for IVIG responders). The authors concluded that resistance to IVIG therapy might be heralded by elevated levels of IL-6, and that IVIG-resistance should contemplate IL-6 in the scoring system.

The contribution of genetics to KS susceptibility and even to responsiveness to IVIG is a matter of great debate. It has been reported that siblings of KS patients have a 6 to 10-fold greater incidence of the disease than the general population [32]. In addition, it has been calculated that the heritability of KS, *i.e.* the ratio of siblings to population incidence of KS, is only slightly lower than that of type 1 diabetes and about four times higher than that of allergic asthma [33]. These and many other findings strongly suggest that genetic factors have a role to play in the occurrence of KS, and in conditioning its severity: studies on genetic characteristics of patients with KS have not, for the time being, precisely identified which kind of genetic markers favour or protect humans from the development of KS. Furthermore, other genetic systems have been associated with resistance to IVIG and development of CAA, including the gene coding for caspase-3, the gene associated with the low affinity receptor of the Fc region of γ-immunoglobulins, and genes related to the transforming growth factor-β signalling pathway [34,35]. Therefore, the existence of genetic factors affecting patients’ responses to treatment with IVIG and risk of cardiac complications is providing further clues to better understanding the overall KS inflammation.

## 6. Conclusions

Children suspected to have KS should receive prompt treatment with IVIG (2 g/kg of body weight as a single dose) within 10 days of illness onset [36]. Many researchers have scrutinised the clinical data and laboratory parameters at onset predicting the risk of CAA [37,38]. Risk factors for CAA are duration of fever longer than two weeks, platelet count, increased acute phase reactants, and age less than five years. No statistically significant difference in the incidence of coronary aneurysms could be observed in consideration of aspirin dosage [39]. Damage to coronary arteries is still a substantial risk for a not negligible percentage of children with KS, mostly in the case they show resistance to IVIG [40]: the identification of this cluster of children at the time of a first clinical assessment might help in discerning those who would benefit from a combined primary treatment with IVIG and corticosteroids. Unfortunately, the available risk scoring systems, which were developed after the analysis of data from Japanese patients, cannot be systematically adopted for patients of different ethnic groups living in other countries. Therefore, the actual identification of non-Japanese patients at higher risk of IVIG resistance is still a challenge.

## Figures and Tables

**Table 1:** The latest scoring systems evaluating non-responsiveness to treatment with intravenous immunoglobulin in children with Kawasaki syndrome: the Egami and Kobayashi scores were primed in 2006, the Sano score in 2007.

**Egami Risk Score [16]**		
*A cut-off point ≥3 identifies patients with Kawasaki syndrome at high risk of being resistant to intravenous immunoglobulin*		
Points		
Age	<6 months	1 point
Days of illness	<4	1 point
Platelet count	<300 × 10^9^/L	1 point
C-reactive protein	>8 mg/dL	1 point
Alanine aminotransferase	>80 IU/L	2 points
**Kobayashi Risk Score [17]**		
*A cut-off point ≥4 identifies patients with Kawasaki syndrome at high risk of being resistant to intravenous immunoglobulin*		
Points		
Sodium	≤133 mmol/L	2 points
Days of illness at initial treatment	≤4	2 points
Aspartate aminotransferase	≥100 IU/L	2 points
Percentage of neutrophils	≥80%	2 points
C-reactive protein	≥10 mg/dL	1 point
Age	≤12 months	1 point
Platelet count	≤300 × 10^9^/L	1 point
**Sano Risk Score [18]**		
*Non-responsiveness to intravenous immunoglobulin is predicted by the presence of at least 2 of 3 predictors in the child with Kawasaki syndrome*		
C-reactive protein	≥7.0 mg/dL	
Total bilirubin	≥0.9 mg/dL	
Aspartate aminotransferase	≥200 IU/L	

**Table 2:** Items evaluated in the different risk scores for Kawasaki syndrome.

Items	Egami Score [16]	Kobayashi Score [17]	Sano Score [18]
C-reactive protein	■	■	■
Age	■	■	
Days of illness	■	■	
Alanine aminotransferase	■		
Total bilirubin			■
Aspartate aminotransferase		■	■
Sodium		■	
Percentage of neutrophils		■	
Platelet count	■	■	

**Table 3:** Differences among the clinical studies for which the latest risk scores for Kawasaki syndrome were formulated.

Clinical Studies	Egami Score [16]	Kobayashi Score [17]	Sano Score [18]
Year of publication	2006	2006	2007
Population	Japanese	Japanese	Japanese
Sample size	320 patients	750 patients	112 patients
Diagnosis of Kawasaki syndrome	Japanese criteria	Revision of the Japanese criteria (5th edition)	Criteria not specified
Diagnosis of coronary artery abnormalities	Not adjusted for body surface area	Not adjusted for body surface area	Adjusted for body surface area (according to de Zorzi’s criteria)
Treatment with intravenous immunoglobulin (IVIG)	Single 2 g/kg/dose within 9 days of illness	1 g/kg per day for 2 consecutive days	1 g/kg per day (within 2 days of illness) for 2 consecutive days
Definition of non-responsiveness to intravenous immunoglobulin (IVIG)	Persistent fever (≥37.5 °C) and fall in C-reactive protein by less than 50% within 48 h after IVIG treatment	Persistent fever (≥37.5°C) lasting more than 24 h or recrudescent fever (after an afebrile period) associated with disease symptoms	Persistent fever (≥37.5 °C) over 24 h after finishing IVIG infusion
Sensitivity	78% ** 61% *	86% **	77% **
Specificity	76% ** 81% *	67% **	86% **

* Referring to the identification of children at higher risk to develop coronary artery abnormalities; ** Referring to the identification of children at higher risk to be non-responders to the administration of intravenous immunoglobulin.
